# Nausea in Specific Phobia of Vomiting

**DOI:** 10.3390/bs3030445

**Published:** 2013-08-15

**Authors:** Yvonne Höller, Mark van Overveld, Heili Jutglar, Eugen Trinka

**Affiliations:** 1 Department of Neurology, Christian-Doppler-Klinik, Paracelsus Medical University, Ignaz-Harrer-Street 79, Salzburg 5020, Austria; E-Mails: heili.jutglar@stud.sbg.ac.at; e.trinka@salk.at; 2 Rotterdam School of Management, Erasmus University Rotterdam, Burgemeester Oudlaan 50, PA Rotterdam 3062, The Netherlands; E-Mail: moverveld@rsm.nl

**Keywords:** nausea, specific phobia of vomiting, emetophobia

## Abstract

Specific phobia of vomiting (SPOV) is a clinical condition with early onset, chronic course and substantial psychosocial impairment due to a rigorous avoidance behavior. A primary symptom which drives patients to consult a medical practitioner is nausea. In this study our aim was to further analyze this symptom of SPOV and examined its role in the development and manifestation of the phobia. We conducted an internet survey in the german SPOV-internet-forum. We calculated a nausea score and grouped participants in a high-and low-nausea group to examine the relationship between nausea and characteristics of the fear of vomiting. In this sample (N = 131), nausea was fairly common in most participants with fear of vomiting. Participants in the high-nausea group had significantly higher ratings of subjective fear and significantly longer duration of fear of vomiting. Additionally, the high-nausea group contained more participants with a body mass index below 19 than the low-nausea group. The present findings suggest that nausea is a core symptom in SPOV which is closely related to intensity of the fear, duration of the fear, and body weight. Future research should investigate if nausea-specific design of treatment could improve therapy outcome.

## 1. Introduction 

Specific phobia of vomiting (SPOV, also known as emetophobia) is still unknown among 29.7% of eating disorder specialists [1]. The rate of clinical practitioners who have never heard about SPOV may even be higher and research on the topic is extremely scarce in the scientific community. Consequently, it is not unreasonable to assume that patients with SPOV may often be misdiagnosed. 

SPOV typically begins in childhood and leads to considerable impairment [2]. Patients with SPOV avoid situations which may increase their risk of vomiting themselves (e.g., eating certain food, traveling, going to an amusement park, meeting ill people, pregnancy...), seeing someone vomiting (e.g., events where guests drink alcohol, meeting pregnant women, places where children play...) [2,3] or vomiting in the presence of others [4]. Hence, patients avoid situations with a high perceived threat value of vomiting. Vomiting cues lead to imagination of worst-case scenarios and activation of autobiographical memories, which in turn maintain the fear of vomiting [5,6]. By consistently avoiding these situations, such biased and catastrophical scenarios about the threat-value of a situation remain uncorrected. 

Moreover, avoidance behavior related to these worst-case scenarios may complicate matters for clinicians in the diagnostic process. A common avoidance behavior in SPOV is food restriction [7], which could potentially lead to misdiagnoses of anorexia [8] or phagophobia [9]. Avoidance behavior of social meeting places (*i.e.*, restaurants) due to a fear of vomiting in the vicinity of others, may lead to misdiagnoses of social phobia or agoraphobia [10]. 

It is therefore important to find distinguishing characteristics of SPOV. An important and potentially distinctive feature of SPOV which could support accurate diagnoses is the occurrence of nausea. Nausea is defined as a discomforting and unpleasant sensation related to the gastrointestinal system. Prior research shows that nausea is indeed regarded as a troublesome symptom in patients which gravely impacts the patients quality of life (e.g., [11]). Interestingly, approximately 10% of the cases which report to (Australian) physicians with complaints of nausea remain undiagnosed [12]. Klonoff *et al.* [13] already provided a description of fear of nausea which was closely related to current definitions of fear of vomiting, in line with the idea that nausea may be particularly relevant to the etiological processes in SPOV. Further, nausea correlates with anxiety [14] and gastrointestinal complaints appear to be significantly associated with a number of anxiety disorders [15]. In addition, while nausea or gastrointestinal symptoms are acknowledged as secondary symptoms in social phobia [15,16] they could play a more crucial and central role in the development of SPOV. Recent work showed that patients with SPOV demonstrate higher levels of disgust propensity (*i.e.*, tendency to experience disgust) and disgust sensitivity (*i.e.*, tendency to evaluate the experience of disgust as a negative experience) compared to a control group without SPOV-complaints [17]. Hence, SPOV could be interpreted as fear of highly aversive, physical cues (*i.e.*, nausea) which are identified as a potential precursor of the catastrophic scenario of vomiting. 

If nausea is indeed relevant for the development of SPOV, nausea could be the predominant symptom perceived by patients with SPOV. According to Boschen [18] nausea in SPOV is due to an increased vulnerability to gastrointestinal anxiety symptoms. Davidson *et al.* [19] reports a higher scoring on the Internal Locus of Control Scale with regard to both general and health-related issues in patients with SPOV. Thus, patients regard nausea and vomiting as being within their influence and try to control these circumstances by rigorous avoidance behavior. Ironically, the more they pay attention to gastrointestinal symptoms, the more likely it becomes that they may perceive nausea. Fearing nausea as a precursor of vomiting leads to selective attention and hypervigilance to gastrointestinal symptoms. Boschen [18] described these cognitive aspects of SPOV. Minimal signs of digestion activity can be catastrophically misinterpreted as indicator for an upcoming nausea, leading to increased anxiety which, in turn, leads to nausea per se [20]. These self-fulfilling fears are not only easily triggered, but also occur in a vicious circle. The more nausea the patient experiences, the more fear raises. Indeed, fear leads to increased hypervigilance and, thus, to stronger feelings of nausea. 

Because nausea is possibly a very important symptom in SPOV, the major aim of this study was to further describe and delineate the role of nausea in fear of vomiting and to investigate whether nausea is indeed a core symptom. Hence, we addressed the following research questions: 

First, we examined whether fear of vomiting was associated with increased levels (and/or a history) of nausea. Therefore, we asked whether participants experienced nausea, asked about the frequency and duration of nausea attacks and asked specific questions to establish how disturbing nausea actually was to the participants. Second, we examined the role of nausea in the course of development of fear of vomiting. Thus, we investigated the onset of nausea in relation to the onset of fear of vomiting. Additionally, we explored whether nausea was associated with core features of the fear, such as locus of fear, intensity of fear, and duration of fear. Third, we were interested in how participants handled nausea, whether they consulted a physician and whether they had any theories about the causes of nausea. Finally, the fourth aim of the study was to investigate whether nausea may complicate the diagnostic process. Therefore, we examined whether higher nausea levels were associated with body weight and eating behavior. This could potentially lead to misdiagnoses of anorexia, for example. At the very least, it would indicate the great difficulty in establishing proper diagnoses when not taking SPOV into account when nausea is a predominant feature in the clinical image of a patient. 

## 2. Experimental Section

### 2.1. Ethics

The Ethics Commission Salzburg/Ethikkommission Land Salzburg was consulted. This Commission rated an internet survey as not being legally bound to be subject to approval. Therefore, the study was just submitted for the attention of the Internal Review Board of the University Clinic of Neurology, Salzburg. The study was designed and carried out according to the ethical requirements as stated in the Declaration of Helsinki. 

### 2.2. Study Population

Participants were recruited at the German internet forum www.emetophobie.de [21]. Therefore, the participants were german speaking and most likely resided in German-speaking countries (e.g., Germany, Austria, Switzerland, Luxembourg, and Liechtenstein). Compiled questionnaires were received from 145 subjects between October 2007 and February 2008. There were late responses from 9 subjects between March 2008 and June 2009. In this sample (N = 154), there were 6 men. 

We selected subjects meeting the DSM-IV-criteria [22] for specific phobia according to their answers in the questionnaire. Participants had to give adequate answers (in parenthesis) to the following questions:

1. “Are you afraid of vomiting? (yes)” AND
2. at least one of the following:
  - “Does the fear of vomiting prevent you from leading a normal life? (yes)”
  - “Do you think you eat normally, as other people do? (no)” AND “Why do you eat differently? (afraid of vomiting)”
  - “Do you abstain from certain dishes because of the emetophobia? (yes)”
3. AND “Are there other things that you avoid because of the fear of vomiting, apart from certain dishes? (yes).”

According to these criteria, 23 participants had to be excluded, resulting in a sample of N = 131. Criterium 2 was distributed as follows: 96.9% participants were impaired in their everyday life because of fear of vomiting, 80.4% (n.a. 26%) indicated an impaired eating behavior, and 61.3% (n.a. 1.5 %) refused eating certain dishes because of the fear of vomiting. 

### 2.3. SPOV Survey

The questions for the survey were designed by the authors. The resulting questionnaire included 36 items and additional questions about the participants’ demographics. It was designed to measure emetophobic complaints. The questionnaire included the items used by Lipsitz *et al.* [2]. Additionally, several new items were constructed to examine the circumstances and consequences of nausea as well as the participants’ eating behavior. A 4-point-Likert-scale (“strongly agree”, “partly agree”, “partly disagree”, “strongly disagree”) was used whenever it was applicable. 

In the present manuscript, not all results on all items are reported because some of them ended up being uniformative, or would not fit thematically to the nausea-specific topic (e.g., we asked participants if they received psychotherapy). The items from which information was extracted for this manuscript were translated from German to English and are attached in the supporting information. 

### 2.4. Procedure

Participants were recruited to voluntarily complete the questionnaire by advertising in the online forum and support group www.emetophobie.de [21]. The advertisement included a link to download the questionnaire and an e-mail address indicating where to send the completed questionnaire. The survey took about 20–30 min to complete. There was no remuneration for participants. The questionnaires were processed anonymously.

### 2.5. Statistics

Data was entered in IBM-SPSS Version 18. We report valid percent and, if applicable (> 0), the percent of participants without an answer (“no answer”, n.a.) in the respective question. That is, the valid percentage was calculated on the sample excluding the proportion of participants without an answer. 

We calculated the median of the following questions: (a) “I suffer from nausea”, “You feel so nauseous... (b)...that you think you won’t stand it any longer”; (c) “...that you nearly have to vomit?”; (d) “Does this nausea interfere with your routine duties?”. This median was our composite nausea score (n.a. in 11 participants because of missing answers). Next, we grouped the sample into participants with a composite nausea score “strongly disagree” or “partly disagree” (low nausea group, N = 80) and participants with a composite nausea score “strongly agree” or “partly agree” (high nausea group, N = 40). 

To examine the relationship between nausea and core features of the fear we calculated two Mann-Whitney-U tests with the nausea group (low/high) as the independent variable and (a) fear-rating and (b) duration of fear as the dependent variables. Additionally, we calculated a chi-square cross tabulation for nausea group and locus of fear (self, others, both), as well as a Kruskal-Wallis test with locus of fear as the independent variable and fear-rating as the dependent variable. 

We were interested in associations between nausea levels and body weight and/or eating behavior. Therefore, we tested the relationship between food avoidance and nausea with a chi-square cross tabula­tion for nausea group and food avoidance (yes/no). To examine the relationship between nausea and body weight, a chi-square test was calculated with the variables nausea-group and BMI group (underweight *vs.* pooled normal weight + overweight; normal weight = 19–25; the overweight group consisted of 8 participants, and was therefore pooled with the normal weight group). In addition, we examined if BMI was influenced by eating-related avoidance behavior by calculating a One-way ANOVA with eating related avoidance behavior (yes, no) as the independent variable and BMI as the dependent variable. 

## 3. Results and Discussion

### 3.1. Results

#### 3.1.1. Demographical and SPOV Characteristics

The demographic data and characteristics of SPOV in our sample of participants with fear of vomiting (N = 131) are listed in Table 1. The majority of the participants were young women (96.6%) with a mean age of 26.47 years (SD = 6.7). Fear of vomiting generally started early, in childhood (M = 9.5 years; SD = 6.4). Locus of fear was in most participants both self and others, but with a higher number of participants reporting more fear in public than in private situations. 

Participants indicated that with respect to vomit they were most afraid of the gag feeling (83.8%), the sight of vomit (72.3%), the sound (71.5%), the smell (66.1%), being disgusted of themselves (33.6%), the fear of suffocating (37.3%), fear of losing control and/or helplessness (14.2%), and the taste (4.8%). 

**Table 1:** Demographic data and characteristics of SPOV in the sample.

Age in years (M, SD, range)	M = 26.47; SD = 6.695; range = 16–47	
female	96,9%	valid N = 127
**begin fear of vomiting (M, SD)**	M = 9.5 y; SD = 6.4 y	
**rating of fear** (%, SD)	86.8%; SD = 15.6%	
**locus of fear**	**percent %**	**valid N**
self	35.10%	46
others	8.40%	11
both equally	56.50%	74
public	67.90%	89
private setting	7.60%	10
both equally	24.40%	32

#### 3.1.2. Nausea

The majority of the participants reported to suffer from nausea (80.9% partly/strongly agreed; n.a. 7.5%). Nausea was intermitted by breaks in 76.7% of the participants while 23.3% described no breaks, that is, a permanent nausea (n.a. 11.5%). Participants reported to suffer from nausea daily (43%), one or more times per week (30.6%), 1–3 times per month (17.4%), or less than once per month (9.1%; n.a. 7.6%). Thus, nausea occurred at least once per week in most (73.6%) participants.

Nausea usually lasted for 30 min or shorter in 35.8% of the participants, 23.3% indicated a duration of 2 h, 15.8% reported a duration of 4 h, and 18.3% suffered from longer lasting periods of nausea. The duration varied in 6.7% of the cases (n.a. 8.4%). 

The relations between the begin of fear of vomiting and the begin of nausea differed among participants. In 46% of the participants, nausea and fear of vomiting started at the same age. There were 44.4% of the participants who reported that fear of vomiting started earlier than nausea. In these participants, nausea began on average 9.68 years (SD = 5.64 years) after the begin of the fear. Further 8% of the participants indicated that nausea began before the fear of vomiting. In this subgroup, nausea started on average 2.67 years (SD = 1.32 years) before fear of vomiting (13% did not report the age of the begin of nausea; 0.8% did not report the begin of the fear of vomiting). 

The majority indicated that nausea interfered with their routine duties (64.8% partly/strongly agreed; n.a. 4.6%). 

Almost half (45%) of the participants reported that nausea was so prominent that they nearly had to vomit. Another 17.8% of participants indicated that the nausea was so disturbing that in certain situations they would prefer vomiting over bearing the nausea. However, 98.3% of the participants did not report actually vomiting whenever they suffered from nausea (n.a. 5.3%). In sum, nausea appeared to be a common and prominent symptom of SPOV. 

Table 2 indicates the results of the statistical tests involving nausea group, fear-rating in %, duration and locus of fear. 

**Table 2:** Comparison of nausea groups, fear, locus of fear, and duration of fear

Comparison	Test name	Test value	df	*p*-value
locus (i) *vs.* fear (d)	KWH	χ^2^ = 1.29	2	0.524
nausea (i) *vs.* fear (d)	MWU	U = 1066	120	0.002
nausea (i) *vs.* duration (d)	MWU	U = 1044	117	0.008
nausea *vs.* locus	chi-square	χ^2^ = 3.28	2	0.194

Results of tests on nausea group, fear-rating in %, duration and locus of fear. i = independent variable; d = dependent variable; KWH = Kruskal-Wallis-H-test; MWU = Mann-Whitney-U-test.

The high nausea group demonstrated higher fear levels and a longer duration of fear compared to the low nausea group. 

Most participants (81.3%) had theories about the situations/circumstances which provoked/caused nausea (18.7% had no theories, n.a. in 6.1%). The frequencies of the proposed situations/circumstances attributed to nausea are shown in Table 3 and suggest that in addition to physical nuisances (*i.e.*, eating specific food), psychological factors like stress and health-concerns are assumed to be associated with experiencing nausea. 

**Table 3:** Situations/Circumstances which were assumed to provoke/cause nausea by the participants.

Situation/Circumstance	%
stress	67.2
having eaten certain food	52.5
health-related	52.3
illness	46
events at work	43.1
events in relationships	41.6
events in the family	35.9
world affairs	14.2
other situations	43.5
n.a.	19.8

As a consequence of nausea, 78% of the participants have consulted a medical practitioner at least once (n.a. 3.1%). A medical practitioner was consulted by 26.5% of the participants once or twice, 40.8% were in consultation 3–10 times, 32.7% more than 10 times (n.a. 25.2%). The outcome of the medical consultation was no diagnosis in 13.5%, a physical diagnosis in 6.3%, a psychic cause for nausea was diagnosed in 50%, and 30.2% were diagnosed with both, physical and psychic causes (n.a. 26.7%). 

The diagnoses were irritable bowel syndrome (20.5%), anxiety disorders (11.9%), gastritis (8.8%), and eating disorders (5.5%). Single participants named depression, somatisation, psychosomatic symptoms, allergy, fungal infection of the intestine, among others (n.a. 46.6%) 

#### 3.1.3. Eating Behavior and Weight

We calculated the body mass index (BMI) based on self-reported height and weight. The overall mean is 20.3 (SD = 3.2) in a range of 13.8 to 34.5. Normal weight (BMI 19–25) was found in 56.5% of the participants. A BMI below 19 (underweight) was found in 37.4% of the participants and a BMI over 25 (overweight) was observed in 6.1% of participants. About half (49.6%) of the subjects was satisfied with their weight, 23.7% found themselves too thin, and 26.7% found themselves too corpulent. Among the 38.9% subjects with a BMI below 19, 59.2% found themselves too thin, 32.7% were satisfied with their weight, and 8.2% (N = 4) found themselves too corpulent. 

Of the underweight-participants, only 16.1% (N = 8, 4 of them anorexia) reported that an eating disorder had been diagnosed by a specialist. In the sample of subjects with a BMI below 19, the majority (91.8%) indicated to suffer from nausea to some extent (partly/strongly agree). 

According to pearsons chi-square test the distribution of high-and low-nausea levels differed between the two BMI-groups (underweight, pooled normal weight+overweight; χ^2^(2) = 3.91; *p* = 0.048). The results show that a high number of nauseous participants are observed in the underweight group and a low number of nauseous participants in the overweight group. 

Next, we examined whether patients with SPOV avoid food and the causes of an eventual food avoidance. Compared to 80.4% of participants with abnormal eating behavior because of fear of vomiting, 19.6% of the subjects reported an abnormal eating behavior because of the fear of gaining weight. Certain food was avoided by 61.2%, 38.8% did not avoid food (1.5% n.a.). The cause for avoiding food was the fear of vomiting because of eating certain food in 98.3% of the participants. Among the most frequently avoided food were raw eggs or dishes containing raw eggs (19.1%), raw meat (15.2%), fish (14.4%), milk and dairy products (12.8%), alcohol (10.3%), fat or fatty food (9.6%), and fast-food (3.2%). Food avoidance did not differ between participants in the high and low nausea group (χ^2^(1) = 1.01; *p* = 0.314). 

As a consequence of abnormal eating behavior 19.6% affirmed suffering from weakness in their job or free-time, 18.1% reported they could not eat with their family and had to eat separately, 34.3% avoided restaurants, 33.5% avoided invitations to come over to eat (n.a. 32.8%; N = 43; of whom 34 reported normal eating behavior). Single participants reported avoiding eating before they left home, the need for checking the best-before-date or the cleanliness of food. 

A borderline significant effect (F(1,127) = 3.33; *p* = 0.07) suggests that participants with avoidance behavior may show up with lower levels of BMI compared to participants without avoidance behavior. 

### 3.2. Discussion

In this study we explored nausea in a relatively understudied disorder (SPOV) because we believe that nausea could be the reason why patients with SPOV often happen to be misdiagnosed. As such, it is crucial to get a better understanding of the role of nausea in SPOV. 

The SPOV-characteristics found in our sample were similar to previous findings. Most participants were young women (similar samples were assessed by [2,3,5,6,17,23]), reported an early begin (as in [2]), fear of vomiting was focused on public situations or both, public or private situations but only in a minority on private situations (see also [3]). The locus of fear was the self or the self and others while only a minority of the participants stated that their locus of fear was only directed towards others (similar findings are documented by [2,3,5,6,23]). 

Nausea was very frequent among the participants of this internet survey. Most patients suffered from nausea at least once a week, although nausea usually did not last longer than a couple of hours ([3] found similar results). More than half of the participants report impairment of routine duties due to nausea, although most of them did not vomit when they felt nauseous. This is well in line with Veale *et al.* [6] who reported that the patients’ avoidance behavior may reduce the risk of vomiting. Among the proposed answer categories, subjects chose fearing the gag feeling, as well as the sight, sound and smell of the vomit (as described in [3]). Additionally, participants reported a fear of losing control, which has been discussed previously as an important factor [19]. 

Thus, the examined sample is comparable to earlier studies in patients with SPOV. In the following, we will discuss the main findings of this survey. 

#### 3.2.1. Nausea in the Maintenance of SPOV 

Participants in the high-nausea group not only showed higher subjective-fear-ratings but also a longer course of the fear of vomiting. As such, nausea could be a maintaining symptom of the fear of vomiting as proposed by Boschen [18]. However, the present cross-sectional design does not allow for firm conclusions regarding potential causal contributions of nausea to fear of vomiting. Future research should explore either an experimental approach (e.g., by examining whether nausea inductions are associated with increases in vomiting-tendencies compared to a control group) or via a longitudinal study to establish the role of nausea in the development of SPOV. This is important, as previous research suggested that nausea may be associated with avoidance behavior. Boschen [18] remarks that avoidance of nausea as a precursor of vomiting causes individuals to never experience situations in which mild nausea does not provoke vomiting. Thus, individuals do not learn about the harmlessness of nausea and interpret nausea-similar experiences such as digestive issues as catastrophic situations (see also [6]). Avoidance, in turn, has recently been reported in relation to nausea. Van Overveld *et al.* [17] reported a higher disgust sensitivity in patients with SPOV and argued that this renders individuals more susceptible to avoidance of situations that evoke nausea. This preoccupation with nausea could lead to a higher probability of experiencing nausea, especially if nausea is indeed closely associated with stress. 

Most interestingly for the present context, our findings indicate that it is unlikely that nausea is a symptom which typically develops late in the course of SPOV. Fear of vomiting and nausea did not necessarily become evident at the same time. Some participants may suffer from nausea for a period of time before fear of vomiting arises and vice versa. Yet, our findings support the view that in a certain subgroup experiencing nausea could contribute to processes related to maintenance of phobic complaints. Nevertheless, further research is needed to clarify the direction of the relationship between fear and nausea. 

#### 3.2.2. Clinical Implications of Nausea

Previous work suggests that patients with SPOV are not aware that nausea could be a consequence of their hypervigilance to gastrointestinal symptoms [18,24]. Instead, they attribute the cause of nausea to physical problems [3,24]. In the current sample, there were different theories about the causes of nausea. As reported by Veale *et al.* [3,24], more than half of the sample attributed nausea to physiological causes, but other reasons were also reported, most notably, psychological factors like stress or peoccupations with health and illness. Additionally, participants identified events in their social network (at work, in relationships, in the family) which influence the feelings of nausea. 

According to our results, many patients start a doctor-shopping tour with many consultations, possibly without a satisfying outcome. This may be due to a relative unawareness in internal specialists on the phenomenology of SPOV. Thus, patients with SPOV may mistakenly be diagnosed as suffering from the irritable bowel syndrome or similar physical problems. Even psychologists or — more precisely — eating disorder specialists may not recognize SPOV [1]. Food-related symptoms such as food-avoidance or underweight could lead to a diagnosis of an eating disorder. Our opinion is that medical misdiagnoses could be avoided if more physicians were familiar with SPOV. 

#### 3.2.3. Nausea’s Relationship to Body Weight and Eating Behavior

To demonstrate how nausea could render proper diagnoses difficult, we explored the relationship between nausea and eating disorder complaints (*i.e.*, body weight, food avoidance). Patients with SPOV can be underweight because of their disordered eating, which is a different situation than being underweight because of an eating disorder [24]. Our results support the view that food restriction is common among patients with SPOV. Food restriction can occur according to volume, in certain situations (e.g., restaurants), at times of stress (e.g., when a patient with SPOV meets someone being sick and is thereafter afraid of being contracted with stomach flu), and patients can also restrict the type of food [7]. The resulting lower intake of calories has the potential to cause underweight. 

Due to low body weight and abnormal eating behavior patients happen to be misdiagnosed as anorectic [8]. In our survey, nausea coincided with lower BMI-levels, suggesting that nausea is associated with food avoidance. Most interestingly, nausea was significantly asymmetrically distributed between BMI groups with a very high number of nauseous participants in the underweight group. Further, a borderline significant difference was observed between participants with and without eating-related avoidance with respect to BMI. It is possible that nausea is related to weight loss in SPOV more directly than food avoidance is. However, this relationship between food-avoidance and BMI may have been stronger if we had asked about food volume restriction. Presently, we do not know if the relationship between nausea and BMI is due to lower food-intake because of nausea, or if nausea (stomach discomfort) is caused by food-restriction. 

Yet, a comorbidity with anorexia nervosa was reported by a small subgroup (4 patients) of the present sample. The findings indicate that nausea is associated with food-avoidance, which could pose making a differential diagnosis between anorexia nervosa and SPOV a challenging task, and even more so without proper knowledge of SPOV-symptomatology. A helpful way to distinguish patients with anorexia and SPOV could be to check the nature of avoidance. If the avoidance of food is concentrated on high-caloric food, this may support the hypothesis that the patient is anorectic. Alternatively, if the patient would avoid invitations, restaurants, eating under certain circumstances (e.g., before leaving home), food that could be contaminated with bacteria (eggs and raw meat) *etc.*, a diagnosis of SPOV may be more likely. The most important distinguishing question is possibly accomplished by asking patients if they avoid food intake because of fearing weight gain or because they fear vomiting. 

#### 3.2.4. Treatment of Nausea

Increased awareness of the SPOV diagnoses could not only fuel more research on effective treatments of SPOV [18], but could also provide fresh clues to help manage nausea levels in treatments where management of nausea poses a challenging factor and can not be controlled well by current emetogenic agents [25,26]. 

Boschen [18] identified nausea and vigilance to gastrointestinal symptoms as a core moderator in SPOV. Using cognitive-behavioral therapy, he recommends to provoke nausea using *in vivo* exposure (*i.e.*, gradual exposure to vomit and/or nausea cues). Recent findings indicate that such an approach, combined with psycho-education and interoceptive exposure techniques could indeed be successful [27]. However, provoking nausea in patients with external locus of fear may not be necessary. We found a borderline-significant relationship between nausea and locus of fear, suggesting that nausea is eventually higher in participants with an internal locus of fear compared to participants with an external locus of fear. This suggests that it could be wise to take into account the locus of fear for the treatment of SPOV. Case reports or case series about therapies in SPOV focus on fear of seeing others vomiting. As such, the patients’ treatment entails exposing them to video material with scenes of vomiting [20,28] or exposing them to the therapist who pretends to be sick [29]. These treatments were successful, possibly just because the therapists used the most-feared cues. Thus, it could be advantageous to design therapies according to the prominence of nausea and both exposure to internal (*i.e.*, nausea) and external (*i.e.*, restaurant-avoidance) cues. 

#### 3.2.5. Limitations

Several limitations can be observed with respect to the current study. 

First, our questionnaire represented a rough indicator of SPOV-complaints, rather than a more elaborate approach such as the Structured Clinical Interview for DSM Disorders (SCID, [30]). Thus, we can not clinically ascertain the diagnoses of SPOV nor control for comorbidity (as done e.g., by [5,6]. Additional validated/standardized questionnaires could have been given to participants alongside to measure level of anxiety, e.g., the HADS [31] or the BAI [32]. Yet, as described above, our sample characteristics are in line with earlier reported samples of patients with SPOV. Moreover, certain questions in our survey tapped into the DSM criteria. 

Nevertheless, the research could have benefitted from a more standardized measurement approach. In our survey, some of the response options for the items may warrant revision to be able to quantify and interpret information more effectively (e.g., a consistent use of equal Likert-scales as answering options for all items). Unfortunately, the Specific Phobia of Vomiting Inventory (SPOVI; [23]) and the EmetQ [33] were only published after completion of this study. Since these were not yet available yet at the time when we conducted the study, we designed our survey based largely on existing questionnaires (*i.e.*, [2]). 

Second, since we conducted an internet survey, we only included participants who were willing to talk about their anxiety online, only. This may bias the composition of the sample. On the other hand, the use of an internet survey did help to include people who would have had to be excluded in other studies (e.g., [5]) because they were to fearful to come in for an interview or who were not able to face such a situation by some other reason. 

Third, there is no control group. Thus, we can not tell whether higher levels of nausea are common and higher in patients with SPOV compared to individuals without clinical complaints. Similarly, the lack of a clinical control group does not allow firm conclusions that nausea plays a very specific role in SPOV, while it is a general symptom in other anxiety disorders such as social phobia and generalized anxiety disorder. 

## 4. Conclusions

In this study we examined the role of nausea in participants with fear of vomiting. Nausea appears to be a common and frequent symptom in people with fear of vomiting. This symptom is associated with increased levels of nausea, it impairs daily routines, is associated with duration of fear, and is thus perceived as very threatening symptom of the fear of vomit. Nausea is not necessarily a precursor or a consequence of fear of vomiting, so further investigations are needed to model the interactions of fear and nausea during the development of SPOV. Since nausea causes people to consider a physician, it is necessary to increase the awareness for SPOV among medical practitioners, especially internal specialists, to avoid misdiagnoses. This is especially important with respect to evidenced relationships between eating behavior/weight and nausea. 

In summary, the role of nausea in development, maintenance, and clinical manifestation of SPOV may have been underestimated until now. As a consequence, nausea should be paid more attention when designing a treatment. Future research should examine the fear and avoidance of nausea in SPOV, because these factors could be crucial in maintaining the anxiety disorder. 

## Supplementary Files

Supplementary File 1

Supplementary File 2
