# Supplementary material for: Nausea in Specific Phobia of Vomiting
Source: Behav Sci (Basel). 2013 Aug 15;3(3):445–58. doi: 10.3390/bs3030445 (PMC4217587; doi:10.3390/bs3030445)
Supplement: Supplementary File 2 [file behavsci-03-00445-s002.doc]

Behav. Sci. **2013**, *3*, 445–458; doi:10.3390/bs3030445

**OPEN ACCESS**

***behavioral***

***sciences***

**ISSN 2076-328X**

www.mdpi.com/journal/behavsci/

Supplementary

Yvonne H¨oller ^1^,*, Mark van Overveld ^2^, Heili Jutglar ^1^ and Eugen Trinka ^1^

^1^ Department of Neurology, Christian-Doppler-Klinik, Paracelsus Medical University, Ignaz-Harrer-Street 79, Salzburg 5020, Austria; E-Mails: heili.jutglar@sbg.ac.at; e.trinka@salk.at

^2^ Rotterdam School of Management, Erasmus University Rotterdam, Burgemeester Oudlaan 50, PA Rotterdam 3062, The Netherlands; E-Mail: moverveld@rsm.nl

* Author to whom correspondence should be addressed; E-Mail: y.hoeller@salk.at;
Tel.: +43-662-44833966; Fax: +43-662-44833004.

*Received: 4 June 2013; in revised form: 24 July 2013 / Accepted: 25 July 2013 /*

*Published:*

**Questionnaire**

Please take your time to answer the questions. Your answers will be handled confidentially and all the information is anonymous—so please answer as truthfully as possible!

Rate the following statements of the presented scales by accenting (italic, underlined, shaded, colored…) or highlighting the answer to the question that seems the most appropriate for you. There is also the possibility of choosing more than one option, that is when there are multiple options such as a, b, c..., then you can choose more than one if they are the case.

There’s also the possibility of answering freely, you can identify such questions when you see a dotted line: Question? You can insert your answer in such gaps.

**(1) I suffer from nausea**

strongly agree partly agree partly disagree strongly disagree

since when? Please answer in age when it started:………………………………………………..

have you suffered from nausea permanently ever since it started or were there times when you
felt better?

Permanently with intermissions

if with intermissions, how many where there?.......................................

and how long did these intermissions last in average?...................................

**(2) You feel so nauseous...**

that you think you won’t stand it any longer

strongly agree partly agree partly disagree strongly disagree

that you nearly have to vomit?

strongly agree partly agree partly disagree strongly disagree

that you have to vomit every time

strongly agree partly agree partly disagree strongly disagree

that you sometimes would prefer to just vomit already, to get rid of the nausea

strongly agree partly agree partly disagree strongly disagree

**(3) Does this nausea interfere with your routine duties?**

strongly agree partly agree partly disagree strongly disagree

**(4) Do you know the situations that cause the nausea?**

yes no

if yes, which situations are these?

Incidents a) in my family b) at work c) with my partner

d) with my friends e) regarding world affairs f) regarding my health

g) certain types of food

if g), which?

i) fatty dishes ii) sugary dishes iii) spicy dishes

others: …………………………………………………………………………

h) stress i) diseases

j) other triggers: ……………………………………………………………………………………….

**(5) Have you ever been to a doctor, to get yourself and especially your stomach examined?**

yes no

if yes: How often have you been to the doctor?

1 time 2 times 3–5 times 5–10 times more often

what was the diagnosis?

psychic causes physical causes both no diagnose

More precisely: Wich diagnosis? …………………………………………………

**(6) Are you afraid of vomit?**

yes no

if yes, please name your age when this fear first occurred:……………………………….

do you suffer from this fear constantly since it first occurred or where there times when you
felt better?

constantly with intermissions

if with intermissions, how many where there?.......................................

and how long did there intermissions last in average?.................................

Are you more afraid of vomiting yourself or of seeing others vomiting?

self others

Is the fear larger in a private setting (e.g. at home) or in a public setting (e.g. at work, when shopping...)?

private setting public setting

how intensive is the fear of vomit? Please try to express the fear in percent, if

0% no fear at all 30% moderate fear 60% great fear

90% very intensive fear 100% unbearable fear

Please rate here in %: 10 20 30 40 50 60 70 80 90 100

**(7) What are you most afraid of with respect to vomit/vomiting?**

a) the sound b) the smell c) the sight of vomit

d) the gag feeling e) being disgusted of myself f) fear of suffocating

**g) others:…………………………………………………………………………………………………**

**(8) What are you most afraid of: Vomiting yourself or to be confronted with it trough
other people?**

self others both equally

**(9) Does the fear of vomit prevent you from leading a normal life?**

yes no

if yes, which parts of your life are expecially constricted?

a) job b) education c) relationships d) leisure/hobbies

e) nutrition f) independence (being able to be alone, live alone…)

for women: g) I avoid pregnancy JUST because of the fear of the pregnancy related sickness

**(10) How long have you suffered from fear of vomit?**

Answer in years/moths:………………………………………………………………………………..

**(11) How much do you weigh? How tall are you?**

…………………………………..kg ………………………………..m

**(12) Are you satisfied with your weight?**

no, I‘m too skinny yes, I’m just right no, I’m too heavy

**(13) Do you think you eat normally, as other people do?**

yes no

if not: Why do you eat differently?

a) diabetes b) allergies c) diet d) afraid of gaining weight

e) afraid of vomiting f) others:………………………………

if not, where does this fear impair you especially?

1. I lack energy for work and leisure, because I don’t eat enough
2. I can never eat what was cooked, I eat separately
3. I can’t go to restaurants
4. I’m constantly thinking of excuses when someone invites me over to eat
5. others:……………………………………………………………………………………………..

**(14) Do you abstain from certain dishes because of the emetophobia?**

yes no

if yes, which ones?…………………………………………………

**(15) Are you afraid of certain dishes?**

yes no

if yes, afraid of:

a) fat b) sugar c) alcohol d) bacteria

e) food gone bad f) others:………………………….

why?

a) because these dishes will make me fat

strongly agree partly agree partly disagree strongly disagree

b) because these dishes might make me vomit

yes no

**(16) Are there other things that you avoid because of the fear of vomit, apart from certain dishes?**

yes no

if yes, which other things? ……………………………………………………………………………………………………..

**(17) Do you suffer from other psychological diseases apart from the emetophobia?**

yes no

if yes, which diseases are these? Please specify if you assume to have this disease or if it is a diagnosis from a doctor (psychiatrist) or a psychologist!

……………………………………………………………………………………………………………

***Personal Information:***

Age:…………………………..

Sex: male female

Education: School years: a) apprenticeship; b) school; c) university; d) other

Occupation: …………………………………………..

Family: married: yes no; children (number): ………………………………..

Living: a) alone; b) with partner; c) with parents/ other relatives; d) flat sharing

© 2013 by the authors; licensee MDPI, Basel, Switzerland. This article is an open access article distributed under the terms and conditions of the Creative Commons Attribution license (http://creativecommons.org/licenses/by/3.0/).
